# Arp2/3 complex contributes to the actin-dependent uptake of *Aspergillus terreus* conidia by alveolar epithelial cells

**DOI:** 10.1371/journal.pone.0341448

**Published:** 2026-01-28

**Authors:** Natalia Mach, Julien Polleux, Lea Heinrich, Lukas Lechner, Iryna Levytska, Cornelia Lass-Flörl, Susanne Perkhofer

**Affiliations:** 1 Research and Innovation Unit, Heath University of Applied Sciences Tyrol/ fh gesundheit Tirol, Innsbruck, Austria; 2 Institute of Hygiene and Medical Microbiology, Medical University of Innsbruck, Innsbruck, Austria; Leibniz-Institut fur Naturstoff-Forschung und Infektionsbiologie eV Hans-Knoll-Institut, GERMANY

## Abstract

*Aspergillus terreus* is an opportunistic fungal pathogen associated with high mortality rates and intrinsic resistance to amphotericin B. Its ability to persist within host tissues without inducing strong immune responses was suggested to contribute to poor clinical outcomes. The cellular mechanisms underlying *A. terreus* interactions with host cells remain largely unexplored. In this study, we have used a micropattern-based infection model to investigate the early interactions between *A. terreus* conidia and alveolar epithelial cells, focusing on the role of Arp2/3-dependent actin remodeling. This system allows quantitative analysis of conidia-cell interactions under defined spatial conditions. We show that *A. terreus* conidia rapidly bind to micropatterned A549 cell islands, with conidial numbers increasing over time. Conidia were found in actin- and Lamp1-positive vesicles already after one hour of infection. Inhibition of the Arp2/3 complex significantly impaired conidial binding and disrupted the formation of actin-positive vesicles, confirming the essential role of Arp2/3-mediated actin remodeling in early stages of conidial uptake. A subset of conidia was localized to Lamp1-positive phagolysosomes and accumulated over time. Interestingly, we have identified a small but consistent population of Lamp1-positive vesicles decorated with actin structures, potentially resembling actin flashes. These structures were entirely abolished upon Arp2/3 inhibition, indicating active cytoskeletal remodeling at the phagolysosomal interface. Our findings provide the first mechanistic insights into *A. terreus* internalization by alveolar epithelial cells and establish Arp2/3-mediated actin dynamics as a key process in early host-pathogen interactions. This cellular pathway may further contribute to intracellular trafficking and help understand the delayed onset of *A. terreus* infections.

## Introduction

Fungal diseases represent a significant global health burden, particularly due to emerging antifungal resistance and high mortality rates among immunocompromised populations [1]. Currently, over 600 fungal species were found to cause human infections. Global estimates based on data from 120 countries suggest that over 2.1 million people develop invasive aspergillosis each year, most commonly in the context of chronic obstructive pulmonary disease, intensive care treatment, lung cancer or haematological malignancies, with a crude annual mortality of about 1.8 million cases (85.2%) [2]. Chronic pulmonary aspergillosis is estimated 1.84 million new cases per year and roughly 340 000 deaths (18.5%) [2]. *Aspergillus terreus* is an opportunistic fungal pathogen that causes invasive infections with high mortality, particularly in immunocompromised patients [3,4]. Although less frequently isolated than *Aspergillus fumigatus*, *A. terreus* is responsible for up to 15% of invasive bronchopulmonary aspergillosis (IBPA) cases and is associated with poor clinical outcomes [5–8]. A major challenge in treatment is the intrinsic resistance of *A. terreus* to amphotericin B, which limits therapeutic options and complicates prophylaxis in high-risk patients [9–12].

Upon inhalation, *Aspergillus* conidia are typically recognized and internalized by phagocytic cells through a well-coordinated mechanism involving Dectin-1, mannose receptors, Toll-like receptors as well as complement-mediated opsonization [13–16]. Once bound, these interactions trigger the engulfment of the conidia into phagosomes, which then fuse with lysosomes to form phagolysosomes where the pathogens are supposed to be degraded [17–19]. The internalization and subsequent acidification of phagolysosomes are critical for the destruction of most fungal pathogens. However, *Aspergillus* species have developed mechanisms to resist or evade immune-mediated destruction, including melanin-mediated masking of immunogenic epitopes and inhibition of phagolysosome acidification [20,21]. In contrast to *A. fumigatus*, which produces DHN-melanin, *A. terreus* synthesizes Asp-melanin, a non-canonical pigment that protects conidia from environmental stress and hampers phagocytosis by soil amoebae [22]. Previous studies demonstrated that *A. terreus* conidia has a distinct immune evasion strategy compared to *A. fumigatus*, characterized by prolonged dormancy within the phagolysosomes of macrophages [6,17,23]. *A. terreus* conidia were shown to be rapidly phagocytosed, but remain dormant and viable within mature, acidified phagolysosomes without germination, thus avoiding immune activation [17,23]. In contrast to *A. fumigatus*, *A. terreus* conidia did not inhibit phagolysosome acidification and were not inactivated by low pH [17,20]. Together, these features of *A. terreus* are characterized as a dormancy-based “sit and wait” strategy likely contributing to chronic infection and poor clinical outcomes [6,17].

Previous studies showed that lung epithelial cells, even being classically non-phagocytic cells, internalize *A. fumigatus* conidia using the F-actin- and Arp2/3-dependent phagolysosomal pathway [24–28]. A study by Slesiona et al found swollen conidia, but no germlings in epithelial lung cells of leucopenic mice infected with *A. terreus* [6]. However, despite similarities in infection models, the precise cellular mechanisms of alveolar epithelial internalization, especially those involving cytoskeletal dynamics in response to *A. terreus*, remain unclear.

In our study, we used a micropattern-based infection model recently developed in our laboratory [29] to investigate in detail Arp2/3-dependent internalization of *A. terreus* conidia into phagolysosomes of alveolar epithelial cells. In this model, extracellular matrix proteins are micropatterned onto PEG-coated coverslips to confine cells to adhesive islands of defined size and geometry, creating multicellular microdomains that can be analyzed individually. This approach enables controlled cell numbers, standardized spatial organization, and improves visualization compared with conventional monolayer culture [29]. Our results provide novel insights into *A. terreus*-specific mechanisms of epithelial uptake and intracellular trafficking of conidia.

## Results

### Binding of *A. terreus* conidia to micropatterned cells

**Fig 1:**
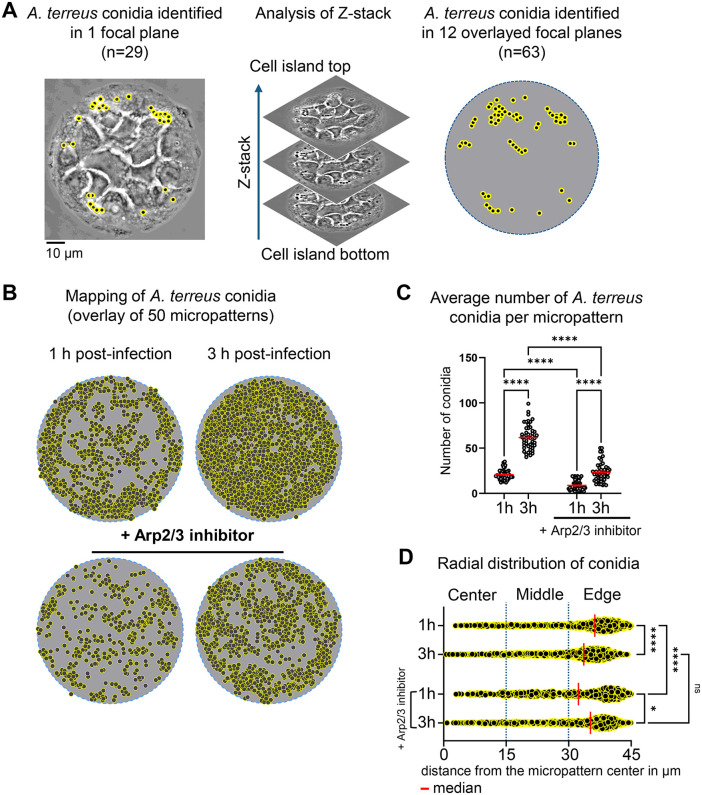
Arp2/3-dependent adhesion of *A. terreus* conidia to micropatterned alveolar epithelial cells. **(A)** Phase contrast image of A549 cells grown on a circular fibronectin-coated micropattern (85 µm diameter), forming a multicellular island. Shown is conidial identification (black dots with yellow circles) in a single focal plane versus 12 overlaid planes of Z-stack after infection with *A. terreus* conidia. **(B)** Computational mapping of conidial positions within 50 overlayed micropatterns. Each dot represents an individual detected conidium across the full Z-stack. **(C)** Quantification of conidial numbers associated with each individual micropattern under control conditions and Arp2/3 inhibition at 1 hour and 3 hours post-infection. Data are shown as scatter dot plots with single data points from 50 micropatterns. Red lines indicate mean ± SEM. ****p ≤ 0.0001. **(D)** Radial distribution of all conidia associated with micropatterned cells, defined as the distance between the geometric center of the conidium and the micropattern center. Three segments are shown (center, middle and micropattern edge); red lines depict median values.

To investigate the interaction between *A. terreus* conidia and alveolar epithelial cells, we used an infection model based on micropatterned substrates, recently established and validated in our laboratory [29]. Confining cells on micropatterns facilitates conidia internalization into phagolysosomes as compared to other cellular models, such as a large-scale cellular monolayer [29]. It also allows accurate mapping and quantification of conidia-cell interactions using an overlay of multiple identical micropatterns. Specifically, we cultured A549 epithelial cells that stably express phagolysosomal marker Lamp1-NeonGreen on fibronectin-coated circular micropatterns with a diameter of 85 µm. Under these conditions, cells consistently formed well-defined multicellular islands containing approximately 15–20 cells per micropattern (Fig 1A). Cells were further infected with dormant conidia of clinical isolate *A. terreus* sensu stricto for 1 and 3 hours. Infection resulted in conidia binding specifically to cellular islands, without unspecific adhesion observed on the surrounding substrate, confirming the high specificity and reproducibility of this model.

To provide a quantitative basis for further analysis, we first quantified the total number of conidia associated with micropatterned cellular islands at both infection time points. This assessment was conducted using multiplane phase-contrast microscopy, enabling quantification of conidia in 12 focal planes of a Z-stack (Fig 1A). More conidia could be identified when analyzing a Z-stack as compared to a single focal plane. Phase contrast visualization included both surface-bound and internalized conidia, without discriminating between these states. Establishing the total conidia count per micropattern served further as an essential baseline metric. The subsequent subcellular localization data were normalized to this number, ensuring accurate comparative analyses. Quantification demonstrated conidia binding to micropatterned cells already at 1 hour post-infection, with a significant further increase in conidia number observed after 3 hours (Fig 1B and Fig 1C, Table in S1 Table). Pre-treatment of cells with Arp2/3 inhibitor CK-666 significantly reduced the average number of conidia associated with micropatterned cells at both the 1-hour and 3-hour time points (Fig 1B and Fig 1C).

Next, we analyzed distribution of conidia associated with individual multicellular islands. All distances from the center of circular micropattern to the individual conidia geometrical center were quantified as distance in µm and plotted in three segments including center, middle and edge of each micropattern (Fig 1D). This analysis revealed that, similar to *A. fumigatus* conidia [29], the majority of *A. terreus* conidia were associated with cells at the outer edge of the micropattern. Application of Arp2/3 inhibitor changed the median value of the distance after 1 hour of infection, but after 3 hours no significant differences were observed, and conidia remained heterogeneously distributed at both time points (Fig 1D).

### Co-localization of *A. terreus* conidia with actin-rich vesicles

**Fig 2:**
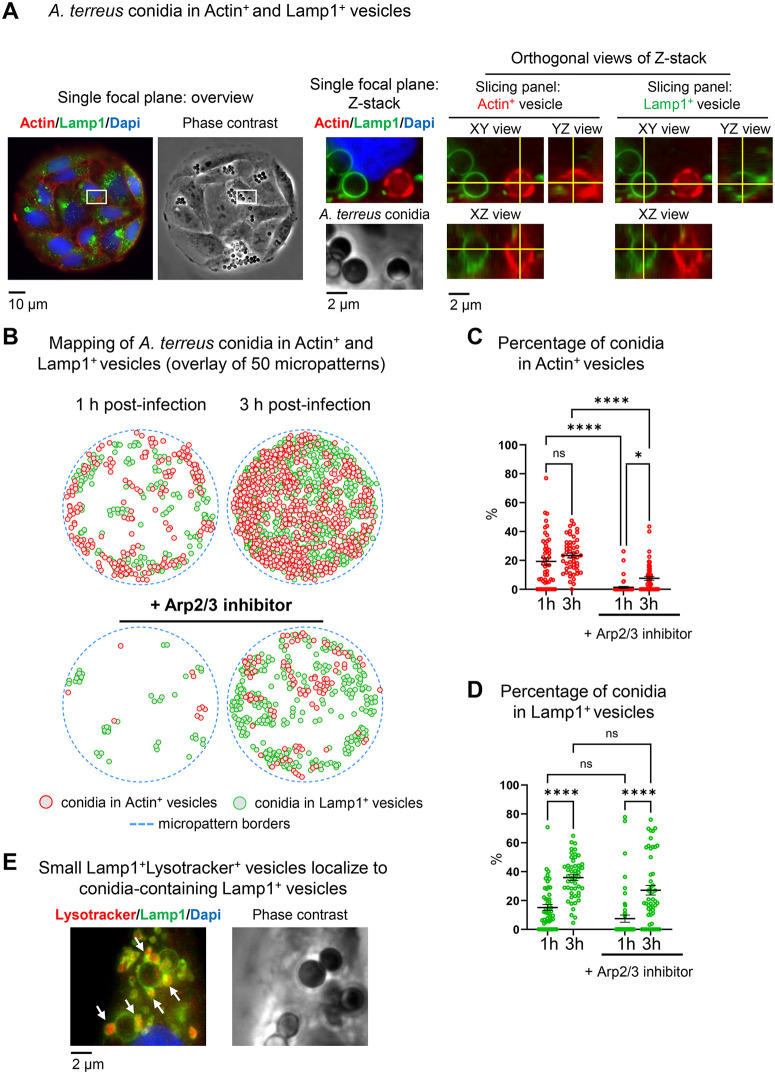
Intracellular compartmentalization of *A. terreus* conidia into micropatterned alveolar epithelial cells. **(A)** Fluorescence images of micropatterned A549 cells expressing Lamp1-NeonGreen (green), stained with LifeAct to visualize actin (red), DAPI for nuclei (blue), and phase contrast image of conidia (grey). Shown are single focal planes and orthogonal views of Z-stack (interval 0.5 µm). The three views are linked by crosshairs (yellow lines). Intersection points on the XY view indicate the slice being viewed in the YZ and XZ panels. **(B)** Computational mapping of Actin^+^ (red circles) and Lamp1^+^ (green circles) vesicle positions within 50 overlayed micropatterns. Each circle represents an individual detected vesicle across the full Z-stack. **(C)** Quantification of the percentage of conidia co-localized with Actin^+^ vesicles. Data are shown as scatter dot plots with single data points from 50 micropatterns. Black lines indicate mean ± SEM. **(D)** Quantification of the percentage of conidia in Lamp1^+^ vesicles. Data are shown as scatter dot plots with single data points from 50 micropatterns. Black lines indicate mean ± SEM. *p ≤ 0.05; ****p ≤ 0.0001; ns, not significant; h- hour. **(E)** Fluorescence images of micropatterned A549 cells expressing Lamp1 (green), stained with Lysotracker to visualize acidified vesicles (red), DAPI for nuclei (blue), and phase contrast (grey). White arrows point on small Lamp1^+^Lysotracker^+^ vesicles localizing near conidia-containing Lamp1^+^ vesicles.

To investigate the role of actin in fungal infection, we applied actin staining and quantified conidia co-localized with actin-rich vesicular structures representing early-stage internalization compartment (Fig 2A). Orthogonal views of Z-stacks supported localization of conidia inside vesicles (orthogonal views in Fig 2A). Here, we use the term ‘internalized’ strictly for conidia enclosed within Actin⁺ vesicles, as visualized in Z-stacks. Given the inherent variability in conidia numbers bound per individual micropattern, normalization to the number of conidia associated with each multicellular island was performed to enable accurate comparative analysis between samples. Our quantitative evaluation revealed that conidial association with actin-rich vesicles (Actin^+^ vesicles) occurred rapidly and efficiently (Fig 2C and Fig 2D). Already after 1 hour of infection, 19.3% of micropattern-associated *A. terreus* conidia were clearly associated with Actin^+^ vesicles. Interestingly, this percentage remained stable and did not significantly increase after 3 hours of infection. Notably, inhibition of the Arp2/3 complex dramatically decreased the number and percentage of conidia associated with Actin^+^ vesicles at 1 hour (1.4%), with some increase detected by 3 hours post-infection (7.6%) (Fig 2C and Fig 2D, Table in S1 Table).

### Internalization of conidia in Lamp1-positive vesicles

To further dissect the subcellular localization of *A. terreus* conidia, we used Lamp1-NeonGreen as a marker representing late endosomal/phagolysosomal compartments (Fig 2B) [29,30]. Similarly to Actin^+^ vesicles, Lamp1 co-localization was quantified relative to the number of conidia associated with individual micropatterns. We observed that localization of conidia to Lamp1-positive phagolysosomes (Lamp1^+^ vesicles) occurred already after 1 hour of infection (Fig 2C and Fig 2E, Table in S1 Table). The percentage of conidia within Lamp1^+^ vesicles significantly increased between 1 hour (15.2%) and 3 hours (35.9%) (Fig 2E). Treatment of cells with Arp2/3 inhibitor substantially decreased the absolute number of conidia found in Lamp1^+^ vesicles (Fig 2C lower panel). However, when normalized relative to all conidia associated with micropattern, no statistically significant differences in the proportion of conidia in Lamp1^+^ vesicles were observed upon Arp2/3 inhibition (Fig 2E). Interestingly, when we tested different sizes of circular micropatterns (60 µm, 85 µm, and 150 µm in diameter), the relative distribution of conidia into Actin⁺ and Lamp1 ⁺ vesicles did not show significant differences (S1 Fig). However, the 85 µm islands yielded more reproducible numbers and smaller fluctuations between individual islands as compared to 150 µm and 60 µm. This may reflect both island size and the spacing between patterns on the UV mask that we currently use.

To further address the compartmentalization of *A. terreus* conidia, we preincubated cells with Lysotracker to identify whereas phagosomal acidification took place. We have found that conidia-containing Lamp1^+^ vesicles were not yet acidified after 3 hours of infection. We have observed multiple small Lamp1^+^ vesicles that were Lysotracker-positive and co-localized with conidia-containing phagolysosomes (Fig 2E, S2 Fig).

### Co-localization of actin foci with Lamp1-positive vesicles

**Fig 3:**
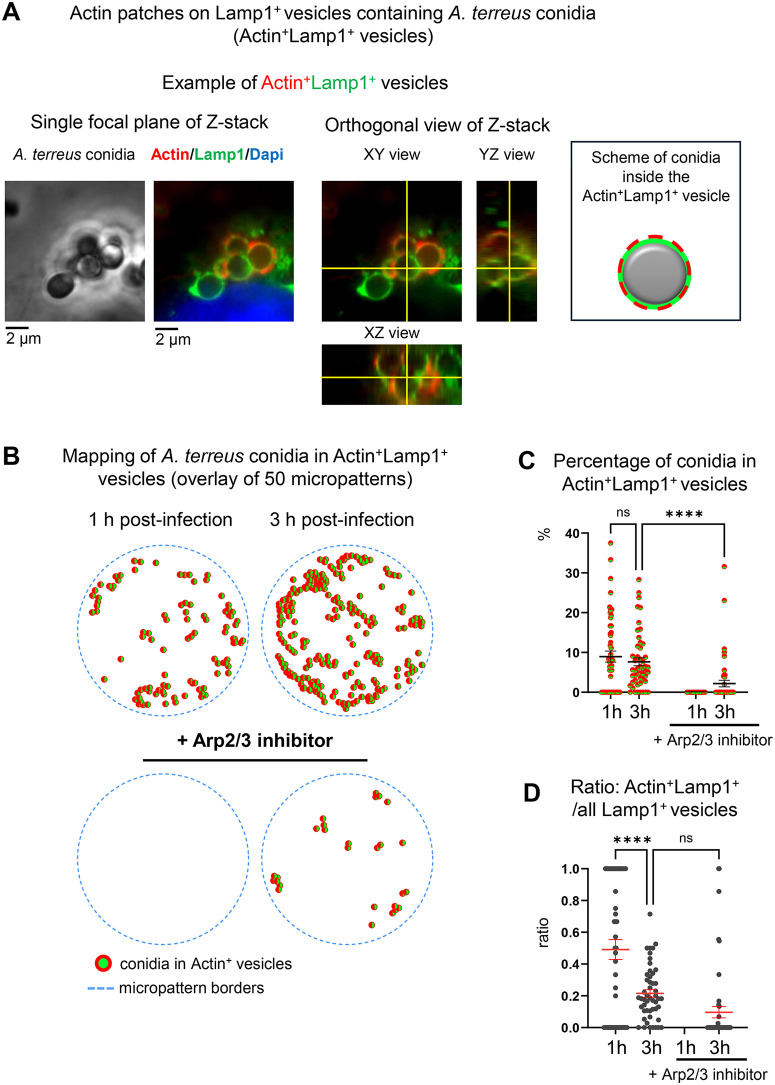
Actin foci decorate a subpopulation of Lamp1^+^ vesicles during *A. terreus* infection. **(A)** Representative fluorescence image showing Lamp1^+^ vesicles with localized actin foci (Actin^+^Lamp1^+^ vesicles). Actin (red), Lamp1 (green) and phase contrast image of conidia (grey) are shown. Single focal planes and orthogonal views of Z-stack (interval 0.5 µm) are displayed. The three views are linked by crosshairs (slicing panels) shown as yellow lines. The three views are linked by crosshairs (yellow lines). Intersection points on the XY view indicate the slice being viewed in the YZ and XZ panels. **(B)** Computational mapping of Actin^+^Lamp1^+^ vesicle positions within 50 overlayed micropatterns. Each circle represents an individual detected vesicle across the full Z-stack. **(C)** Quantification of conidia associated with Actin^+^Lamp1^+^ vesicles. Data are shown as scatter dot plots with single data points from 50 micropatterns. Black lines indicate mean ± SEM. **(D)** Ratio of double-positive vesicles (Actin^+^Lamp1^+^) relative to all identified Lamp1^+^ vesicles. Data are shown as scatter dot plots with single data points from 50 micropatterns. Black lines indicate mean ± SEM. ****p ≤ 0.0001; ns, not significant; h- hour.

While analyzing the localization of *A. terreus* conidia within Lamp1^+^ phagolysosomes, we observed distinct actin accumulations, here defined as “actin foci”, on a subset of Lamp1^+^ vesicles containing conidia (Actin^+^Lamp1^+^ vesicles) (Fig 3A, Table in S1 Table). Actin foci localized on top of Lamp1^+^ vesicles rather than co-localized with it. Actin^+^Lamp1^+^ vesicles were detected in 25 out of 50 analyzed micropatterns already after 1 hour of infection (8.96% of micropattern-associated conidia) and showed no significant change at 3 hours (7.64%) (Fig 3B, Fig 3C and Fig 3D, Table in S1 Table). Treatment with Arp2/3 inhibitor completely abolished actin foci at 1 hour, with only a minimal reappearance at 3 hours post-infection (2.17%) (Fig 3B and Fig 3C). Further, we calculated the fraction (ratio) of Actin^+^Lamp1^+^ vesicles relative to all identified Lamp1^+^ vesicles, revealing a decrease from 0.49 at 1 hour to 0.22 at 3 hours post-infection (Fig 3D). Following inhibitor treatment, the ratio quantification was only possible at the 3-hour time point and was not significant due to a very low abundance of Actin^+^Lamp1^+^ vesicles.

## Discussion

In the present study, we investigated the early interactions of *A. terreus* conidia with alveolar epithelial cells. The primary objective was to elucidate processes of fungal adhesion and subsequent trafficking into Lamp1-positive phagolysosomal compartments. These aspects were previously unexplored for *A. terreus* in epithelial models. Our findings confirm that epithelial internalization of *A. terreus* can be mechanistically dissected and offer new opportunities to explore early host-pathogen interactions in detail.

Our findings revealed rapid adhesion of *A. terreus* conidia to micropatterned alveolar epithelial cell islands, detectable already at 1 hour post-infection. The number of cell-associated conidia increased further by three hours, highlighting ongoing adhesion events. We also identified a central role of the Arp2/3 complex in mediating early interactions between epithelial cells and *A. terreus* conidia. Conidial adhesion was strongly dependent on Arp2/3-driven actin polymerization, consistent with its well-characterized function in supporting phagocytosis [28,31–34]. Inhibition of Arp2/3 led to a pronounced decrease in fungal binding and formation of Actin^+^ vesicles, indicating that actin polymerization is essential for establishing early host-pathogen contacts and initiating internalization. These observations also suggest that Arp2/3 may function as a dual mediator by facilitating both membrane-cytoskeleton coupling during uptake and cytoplasmic remodeling events after pathogen entry. Similar to our previous observations with A. fumigatus on 60 µm multicellular islands, we found that A. terreus conidia were not distributed uniformly across the cell islands but instead were more frequently associated with the micropattern edges than the center. This peripheral enrichment likely results from increased membrane availability and greater cell surface exposure at the edges of the island.

Our study highlights the central importance of the actin cytoskeleton in the internalization of *A. terreus* conidia by alveolar epithelial cells. The rapid association of fungal conidia with actin-rich vesicles underlines actin polymerization as a crucial step in early interactions. In professional phagocytes, Arp2/3-driven actin filament branching was shown to promote membrane protrusions and phagocytic cup formation, necessary for pathogen engulfment [33,35–37]. Our results extend previous findings to *A. terreus* and reveal that alveolar epithelial cells utilize Arp2/3-dependent mechanisms for fungal uptake.

A particularly interesting feature of the intracellular trafficking of *A. terreus* conidia was the differential behavior of Actin^+^ versus Lamp1^+^ vesicle populations over time. The Lamp1^+^ vesicles containing *A. terreus* conidia progressively accumulated between one and three hours of infection, indicating an increased localization of conidia to phagolysosomal compartment. However, our experiments revealed that conidia-containing Lamp1^+^ compartments were not yet evidently acidified within the 3-hour infection time. Acidified Lamp1^+^ vesicles, were observed mainly as small compartments lacking conidia or near conidia-containing vesicles. This suggests that Lamp1 recruitment precedes acidification, and conidia may remain in non-acidified, intermediate compartments during early infection.

Interestingly, the percentage of conidia surrounded by actin remained comparatively stable. These results suggest that actin association is not a cumulative process, but rather a transient interaction that resolves quickly after pathogen internalization. We hypothesize that actin polymerization around internalized conidia is an early and short-lived event, potentially functioning as a dynamic scaffold to stabilize conidia binding and early vesicle formation. We have also observed a subset of Lamp1^+^ vesicles surrounded by distinct actin structures, forming a population of Actin^+^Lamp1^+^ vesicles. These were not independent vesicles but Lamp1^+^ compartments transiently decorated with actin foci, morphologically consistent with actin flashes [38,39]. Actin flashes have been previously reported in phagocytic systems as short-lived, Arp2/3-mediated events that transiently associate with maturing phagosomes [38–40]. The function of actin flashes is not completely understood; they have been shown to modulate vesicle trafficking by controlling the timing of fusion events and/or limiting premature acidification [38,41,42]. In our system, the presence of actin-decorated Lamp1^+^ vesicles at both one and three hours of infection, as well as their strong sensitivity to Arp2/3 inhibition, supports the view that actin flashes in epithelial cells may represent transient intermediates rather than stable compartments. Moreover, the relative increase in Lamp1^+^ localization over time, in the absence of a corresponding increase in the fraction of Actin^+^Lamp1^+^ vesicles, further supports this interpretation. As the proportion of micropattern-associated conidia in Lamp1^+^ vesicles increased between one and three hours, the ratio of Actin^+^Lamp1^+^ to all Lamp1^+^ vesicles declined, suggesting that actin recruitment to Lamp1^+^ phagosomes is temporally restricted and not a default feature of all Lamp1^+^ compartments.

Instead, actin flashes may occur on a subset of phagosomes. Their formation requires active Arp2/3 signaling, since we observed nearly complete loss upon inhibitor treatment. This interpretation remains speculative and will require validation by live-cell imaging approaches.

Interestingly, a recent study has shown that the early endosomal GTPase Rab5c protein is critical for efficient phagosome maturation and LC3-associated phagocytosis in response to *A. fumigatus* conidia but is not required for their initial internalization [43]. This supports the view that uptake and intracellular processing are mechanistically separable. In our study, *A. terreus* conidia remained associated with Actin^+^ vesicles over time, suggesting delayed vesicle maturation. Although we did not directly investigate Rab5 or LC3 pathways, these findings raise the possibility that prolonged actin retention could influence the timing of recruitment of early endosomal and autophagy-related factors. Whether *A. terreus* actively modulates Rab5-dependent signaling or LC3-associated phagocytosis in epithelial cells remains to be evaluated experimentally.

These findings introduce a new factor to the host response to *A. terreus* conidia. In contrast to *A. fumigatus*, which shows minimal actin co-localization under similar conditions ([29] and our unpublished data), *A. terreus* induces a stronger actin response. The presence of Lamp1^+^ vesicles with actin flashes could reflect a fungal strategy to weaken normal degradation pathways, either by delaying phagosome maturation or by modulating antigen presentation mechanisms. However, our data are restricted to early time points and do not directly address conidial viability, dormancy or killing. The observed accumulation of conidia in Lamp1^+^ vesicles over time in untreated cells is consistent with progressive phagosome maturation toward degradative endpoints [24,29,44]. The ability of conidia to reach Lamp1^+^ vesicles even under Arp2/3 inhibition suggests that phagolysosomal routing is at least partially Arp2/3-independent, as described for other microbial pathogens [45,46]. Our results imply that once internalized, conidia can engage alternative trafficking routes.

Using our current micropattern-based model, we could not quantify overall internalization efficiencies, as analyses were normalized to micropattern-associated conidia and not to the initial inoculum. Furthermore, our micropattern-based model is currently not well suited for long-term experiments, because cells cannot be maintained in confined islands for prolonged periods that would be required to follow the fate of internalized conidia (our unpublished observations). Another important limitation is that our experiments were performed in A549 cell line. Future work should examine whether the observed Arp2/3-dependent uptake mechanisms and actin flashes are also present in primary human alveolar cells or other clinically relevant epithelial models.

In conclusion, our study provides the first mechanistic insights into *A. terreus* internalization and intracellular trafficking in alveolar epithelial cells. Our findings suggest an important role for the Arp2/3 complex in cell-conidia interactions. Future work will be required to link these early trafficking events to long-term fungal fate and to evaluate whether targeting of Arp2/3-dependent pathways can be exploited therapeutically.

## Materials and methods

### Fungal strain and growth conditions

The clinical isolate *Aspergillus terreus* sensu stricto (strain no 375) was cultured on Sabouraud 4% glucose agar (Sigma-Aldrich, 84088, Austria), prepared with 40 g/L D (+)-glucose (Sigma-Aldrich, 1.08337, Austria) and 10 g/L peptone (Carl Roth, 8986.1, Germany) and incubated at 37 °C for ten days to ensure conidia maturation. Conidia were harvested by gently flooding the fungal culture surface with sterile spore buffer containing 0.1% Tween (Fisher Scientific, 10113103, Germany) and 0.9% NaCl (Sigma-Aldrich, 1.06404, Austria). The suspension was centrifuged at 4000 rpm for 5 min, the pellet was resuspended in sterile spore buffer and was directly used for infection experiments.

### Cell culture and Arp2/3 inhibition

A549 human alveolar epithelial-like cell line stably expressing the phagolysosomal marker Lamp1-NeonGreen [30] was cultured in RPMI-1640 (Capricorn, RPMI-XA, Germany) supplemented with 10% fetal bovine serum (FBS, Capricorn Scientific, FBS-12A, Germany), 2 µl/ml puromycin, (Carl Roth, 0240.2, Germany), 1% penicillin/streptomycin mix (Capricorn Scientific, PS-B, Germany) and 1% L-glutamine (Capricorn Scientific, GLN-B, Germany). For Arp2/3- inhibition, cells were pretreated with the Arp2/3- complex inhibitor CK666 (150 µM, THP Medical Products, HY-16926, Austria) two hours prior to infection. The inhibitor was maintained throughout the experiment to ensure continuous disruption of Arp2/3--mediated actin polymerization, as established in previous studies [28].

### Micropatterned substrate preparation and cell seeding

Micropatterned substrates were fabricated on PEG-silane-modified glass coverslips using deep ultraviolet (UV) photolithography, as previously described [29,47,48]. Briefly, glass coverslips were incubated at 80 °C under an inert atmosphere in a 1 mM solution of linear PEG-silane (Rapp Polymere, Germany) dissolved in dry toluene for 20 hours, promoting covalent attachment of PEG to glass. PEG-coated coverslips were thoroughly rinsed with isopropanol, methanol, and distilled water, and dried with nitrogen. PEG-coated coverslips were placed on a chromium photomask featuring circular shapes of 85 µm in diameter (Compugraphics, Jena) and exposed to deep UV for 5 minutes (low-pressure mercury UV lamp, 185 nm, Heraeus Noblelight, Germany) at 5 cm distance. The UV exposure locally oxidized the PEG layer, allowing subsequent protein adsorption. Micropatterned coverslips were further incubated at 4 °C overnight with fibronectin (10 µg/ml in PBS, Sigma-Aldrich, 341631, Austria) to promote cell adhesion. After rinsing in PBS, cells were seeded at a density of 1 × 10⁶ cells/mL in RPMI with 1% FBS and incubated for 2 hours at 37 °C. Non-adherent cells were gently removed by washing with pre-warmed PBS. The remaining cells were cultured overnight in the same serum-reduced (1%) RPMI medium to allow proper spreading on micropatterns.

### Infection with *A. terreus* conidia

After overnight incubation on micropatterned fibronectin-coated coverslips, A549 cells were infected with 10^7^ cfu/ml of *A. terreus* conidia. This amount was required to achieve reproducible binding across all islands, we have previously tested 10^5^ and 10^6^ cfu/ml that produced highly variable results (our unpublished data). Before infection, serum-reduced medium was replaced with full-growth RPMI-1640 containing 10% FBS and no antibiotics. Cells were incubated with conidia for either 1 hour or 3 hours at 37 °C under standard cell culture conditions and further processed for fixation. High MOI was

### Fluorescence microscopy

To preserve Lamp1-positive vesicles and actin cytoskeleton, cells were fixed using a “mild fixation” protocol, specifically optimized for these structures [49]. Fixed cells were further incubated with 0.05% saponin (Sigma-Aldrich, 47036, Austria) in PBS for permeabilization and simultaneously stained with EasyProbe ™ ActinRed 555 (FP032, THP Medical Products, Austria) and DAPI (1:20.000, D9542-5MG, Sigma-Aldrich, Austria) at room temperature for 1 hour. Following three PBS washes, samples were mounted on glass slides using Mowiol mounting medium (Sigma-Aldrich C9368, Austria). Lysotracker DeepRed (Invitrogen, L12492, 50 nM) was applied 2 h before infection start and was present during infection and washing steps, following the same mild fixation protocol. Fluorescence images were acquired using an Olympus IX83 inverted wide-field microscope (Olympus Austria) equipped with a UPlanXApo 60 × /1.42 NA objective and 2 × digital zoom. Z-stacks were recorded in parallel using a 0.5 µm step size across all imaging channels (DAPI, FITC, TRITC, and phase contrast PH3), at a resolution of 2048 × 2048 pixels and 16-bit grayscale. Exposure times were 8 ms (DAPI), 120–150 ms (TRITC/FITC), and 60 ms (phase contrast). Image stacks were deconvoluted using Olympus cellSens software with nearest-neighbour settings (50%). For each condition, 50 micropatterned cells from three biological replicates were analysed.

### Image analysis and quantification

Image evaluation was carried out using Fiji software (RRID: SCR_002285). Three trained researchers independently identified and quantified vesicle types by analysing merged fluorescence channels: Actin^+^ (TRITC), Lamp1^+^ (FITC), and Actin^+^Lamp1^+^ (TRITC/FITC co-localization) compartments. The “Point tool” and “Analyze-Measure” functions were used to record the X/Y- positions of fungal conidia and their associated vesicles. Z-stack data were reviewed in parallel to assess cell numbers (nuclear staining with DAPI), total conidial counts (phase contrast), and subcellular localization in relation to actin and Lamp1 fluorescent signals.

### Statistical analysis

Data from three biological repetitions for each experiment and 50 micropatterns were used for each data point. All quantitative data were processed in GraphPad Prism 10.1.2 (RRID: SCR_002798). Statistical tests included descriptive statistics and ordinary one-way ANOVA with multiple comparisons. Data median, mean and standard error of the mean were quantified in GraphPad Prism. Figures were prepared in Fiji and finalized using Adobe Photoshop (RRID: SCR_014199). The statistical significance of the data was verified by p-values. Original data is disclosed in S2 Table in Supporting information.

## Supporting information

S1 Fig Comparison of conidial interactions with cells grown on circular micropatterns of 60 µm, 85 µm and 150 µm in diameter. (A) 1 hour post-infection. (B) 3 hours post-infection. Graphs on the left side depict quantification of average conidial number associated with each individual micropattern (black circles). Red lines indicate mean ± SEM. Quantification of the percentage of conidia co-localized with Actin^+^ (red circles), Lamp1^+^ (green) and Actin^+^Lamp1^+^ vesicles (red/green half circles). Black lines indicate mean ± SEM. Data are shown as scatter dot plots with single data points from 50 micropatterns.
(PDF)

S2 Fig Co-localization of small Lamp1^+^Lysotracker^+^ lysosomes with conidia-containing Lamp1^+^ vesicles. Shown are images of micropatterned A549 cells expressing Lamp1-NeonGreen (green), stained with Lysotracker to visualize acidified vesicle (red), DAPI for nuclei (blue), and phase contrast image of conidia (grey). Image shows maximal projection of 6 sequential images of Z-stack.
(PDF)

S1 Table Summary of quantitative analysis of *A. terreus* conidia interactions with micropatterned A549 cells. (DOCX)

S2 Table Dataset with original data. (XLSX)
