# Supplementary figures and images for: Arp2/3 complex contributes to the actin-dependent uptake of Aspergillus terreus conidia by alveolar epithelial cells
Source: PLoS One. 2026 Jan 28;21(1):e0341448. doi: 10.1371/journal.pone.0341448 (PMC12851495; doi:10.1371/journal.pone.0341448)

Supplementary Figure 1

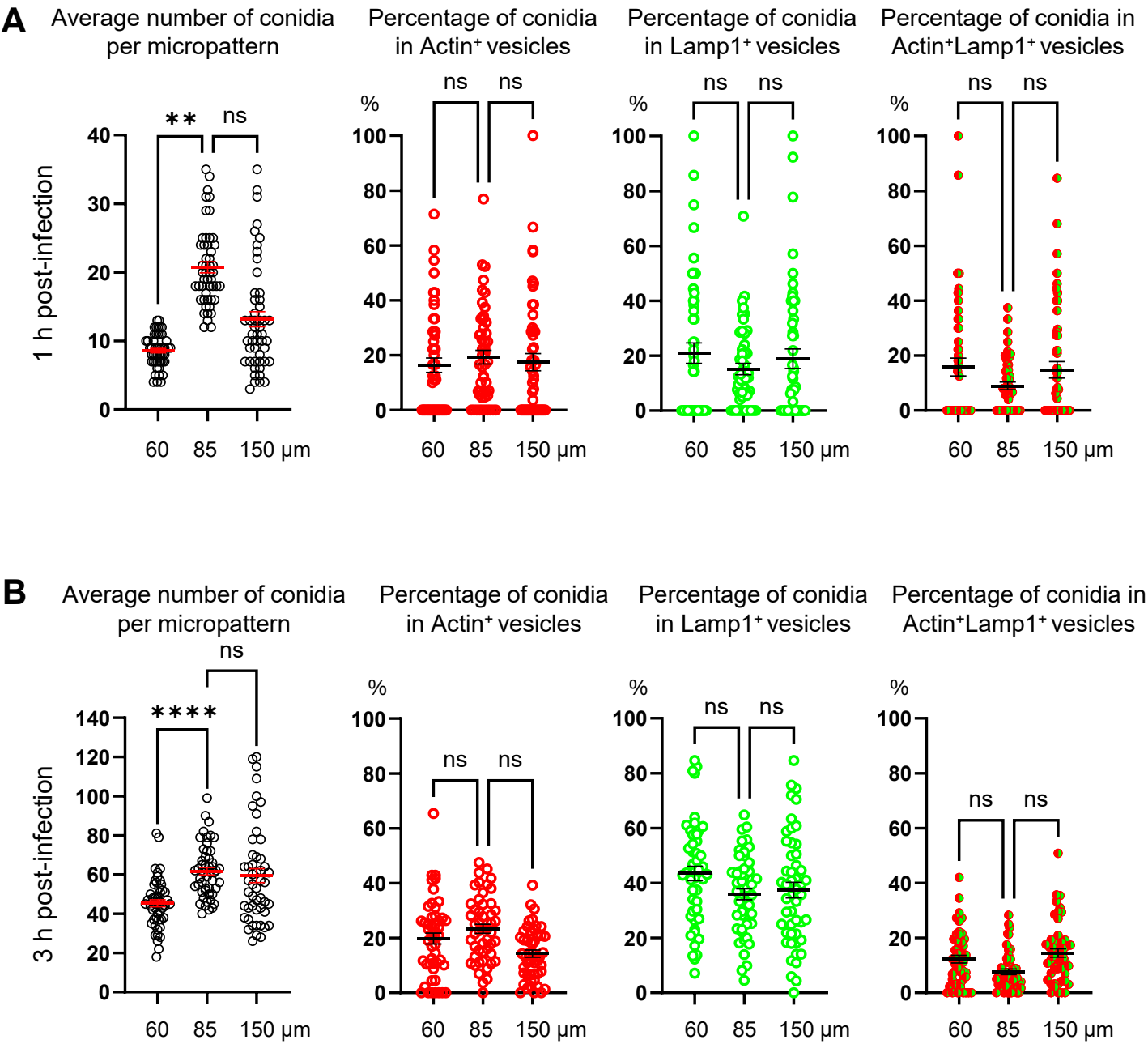

Supplement: S1 Fig — (A) 1 hour post-infection. (B) 3 hours post-infection. Graphs on the left side depict quantification of average conidial number associated with each individual micropattern (black circles). Red lines indicate mean ± SEM. Quantification of the percentage of conidia co-localized with Actin+ (red circles), Lamp1+ (green) and Actin+Lamp1+ vesicles (red/green half circles). Black lines indicate mean ± SEM. Data are shown as scatter dot plots with single data points from 50 micropatterns. (PDF) [file pone.0341448.s001.pdf]
