# Supplementary material for: Arp2/3 complex contributes to the actin-dependent uptake of Aspergillus terreus conidia by alveolar epithelial cells
Source: PLoS One. 2026 Jan 28;21(1):e0341448. doi: 10.1371/journal.pone.0341448 (PMC12851495; doi:10.1371/journal.pone.0341448)

Supplementary Figure 2

Co-localization of small Lamp1$^+$Lysotracker$^+$ lysosomes with conidia-containing Lamp1$^+$ vesicles. Shown is maximal projection of Z-stack

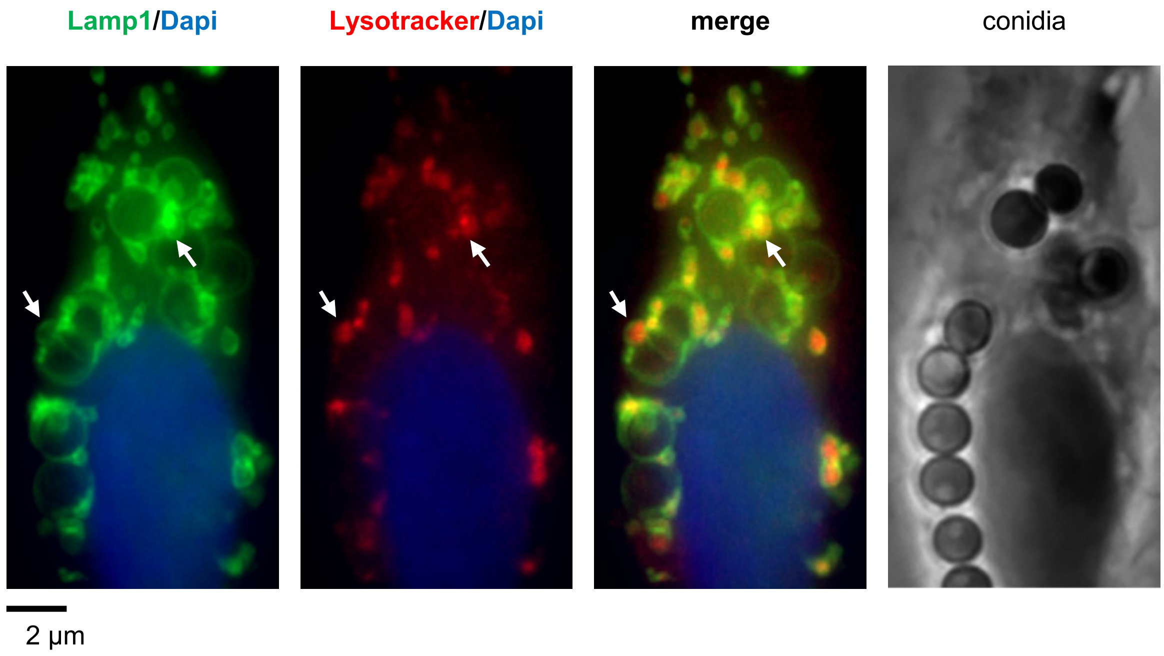

Supplement: S2 Fig — Shown are images of micropatterned A549 cells expressing Lamp1-NeonGreen (green), stained with Lysotracker to visualize acidified vesicle (red), DAPI for nuclei (blue), and phase contrast image of conidia (grey). Image shows maximal projection of 6 sequential images of Z-stack. (PDF) [file pone.0341448.s002.pdf]
