# Supplementary material for: Arp2/3 complex contributes to the actin-dependent uptake of Aspergillus terreus conidia by alveolar epithelial cells
Source: PLoS One. 2026 Jan 28;21(1):e0341448. doi: 10.1371/journal.pone.0341448 (PMC12851495; doi:10.1371/journal.pone.0341448)
Supplement: S1 Table — (DOCX) [file pone.0341448.s003.docx]

Supporting information

**S1 Table. Summary of quantitative analysis of *A. terreus* conidia interactions with micropatterned A549 cells**

| Time post-infection | Conidia in Actin^+^ vesicles | Conidia in Lamp1^+^ vesicles | Actin patches on Lamp1^+^ vesicles (Actin^+^Lamp1^+^ vesicles) | Ratio of Actin^+^Lamp1^+^ to all Lamp1 vesicles | Average number of conidia associated with individual micropattern | Total number  of conidia associated with 50 micropatterns |
| --- | --- | --- | --- | --- | --- | --- |
| 1 hour | 19.30±2.48% | 6.20±1.30% | 8.96±1.38% | 0.49±0.06 | 20.74±0.80 | 1037 |
| 3 hours | 23.40±1.67% | 28.20±1.76% | 7.64±1.00% | 0.22±0.02 | 61.72±1.90 | 3086 |
| 1 hour  (+CK666) | 1.35±0.69% | 7.47±2.52% | 0.00±0.00% | 0.00 | 8.84±0.76 | 442 |
| 3 hours (+CK666) | 7.59±1.40% | 25.0±3.29% | 2.17±0.832% | 0.10±0.04 | 23.14±1.52 | 1157 |
